# Integrated Payment, Fragmented Realities? A Discourse Analysis of Integrated Payment in the Netherlands

**DOI:** 10.3390/ijerph19148831

**Published:** 2022-07-20

**Authors:** Thomas Reindersma, Isabelle Fabbricotti, Kees Ahaus, Sandra Sülz

**Affiliations:** Department of Health Services Management & Organisation, Erasmus School of Health Policy & Management, Erasmus University, Burgemeester Oudlaan 50, 3062 PA Rotterdam, The Netherlands; fabbricotti@eshpm.eur.nl (I.F.); ahaus@eshpm.eur.nl (K.A.); sulz@eshpm.eur.nl (S.S.)

**Keywords:** integrated payment, integrated care, discourse, healthcare policy, alternative payment models

## Abstract

The current models used for paying for health and social care are considered a major barrier to integrated care. Despite the implementation of integrated payment schemes proving difficult, such initiatives are still widely pursued. In the Netherlands, this development has led to a payment architecture combining traditional and integrated payment models. To gain insight into the justification for and future viability of integrated payment, this paper’s purpose is to explain the current duality by identifying discourses on integrated payment models, determining which discourses predominate, and how they have changed over time and differ among key stakeholders in healthcare. The discourse analysis revealed four discourses, each with its own underlying assumptions and values regarding integrated payment. First, the Quality-of-Care discourse sees integrated payment as instrumental in improving care. Second, the Affordability discourse emphasizes how integrated payment can contribute to the financial sustainability of the healthcare system. Third, the Bureaucratization discourse highlights the administrative burden associated with integrated payment models. Fourth, the Strategic discourse stresses micropolitical and professional issues that come into play when implementing such models. The future viability of integrated payment depends on how issues reflected in the Bureaucratization and Strategic discourses are addressed without losing sight of quality-of-care and affordability, two aspects attracting significant public interest in The Netherlands.

## 1. Introduction

Due to demographic and technological changes, an increasing number of people are depending on integrated care. However, the current approach to funding health and social care is seen as one of the major barriers to realizing integrated care [1,2]. Recognizing this, countries have begun experimenting with integrated payment schemes for healthcare and social services [3,4]. Like integrated care, integrated payment assumes many forms—common models are bundled payment or population-based payment [5]. A shared feature of these models is that payment is disbursed to groups of providers. The first is designed to cover episodes of care, certain procedures or (chronic) conditions, whereas the latter provides coverage for well-defined populations [5]. Shifting risk from the payer to provider groups is part and parcel to integrated payment, and sharing this risk between providers in the group consequently increases financial and clinical accountability [6,7]. This is expected to stimulate coordination and integration between providers [8], potentially leading to cost savings and improved quality of care [6]. Integrated care dimensions such as horizontal, vertical, and sectoral integration can also be used to map the coverage of integrated payment [9]. In instances where integrated payment covers and stimulates horizontal integration, with providers performing similar functions (e.g., multiple hospitals), competition is assumed to be fiercer compared to vertical integration, where successive partners in a chain collaborate (e.g., hospital and nursing home) [10]. This is assumed to be further complexified when the scope of integrated payment widens to also cover intersectoral collaboration (e.g., health and social services) [11]. Hence, the development and implementation of such schemes is complex, where giving substance to the key elements constituting such models requires the cooperation of regulators, payers, provider organizations and professionals [12]. Experiments have not yet led to conclusive improvements in terms of cost containment or outcomes [13,14,15].

Despite these issues, initiatives to stimulate, develop, and implement integrated payment schemes remain prominent on the agenda of many countries. In the Netherlands, as in other countries [5], this focus has led to a payment architecture that is characterized by its duality. Various types of healthcare provision in the Netherlands have different reimbursement schemes, such as diagnosis-treatment combinations (similar to diagnosis-related groups) for hospitals, and capitation plus consultation fees for general practitioners (GPs). During the past decade, integrated payment models have been introduced on top of this basic framework. The duality created through multiple payment regimes introduces administrative challenges and conflicting financial incentives [16]. For instance, critics argue that maintaining current fee schedules as the basic framework and building layers of alternative payment models on top will not “fix a broken system” [16] (p. 5). The (further) development of payment policy should be devoted to striking a delicate balance between incentives [17]. Currently, whether the duality can thrive is contested as is whether the basic framework will be replaced by or continues to co-exist with other, integrated, payment schemes in the near future.

Our aim is to understand how the Netherlands arrived at this dual payment structure through analyzing the discourses of underlying values and beliefs regarding integrated payment. The primary objective is to determine which discourses predominate, how they have changed over time, and also differ among the key stakeholders in healthcare, to ultimately gain insight into the justification and viability of continuing to implement integrated payment in the future. Further, this will provide an understanding of why integrated payment schemes are pursued despite the lack of clear-cut evidence on their effectiveness [4,12,13,14,18]. The Netherlands is an interesting setting for such a discourse analysis given its neo-corporatist style of policymaking, characterized by extensive consultations with a wide array of stakeholders [19], and a system based on managed competition [20] with both insurers and providers competing with similar organizations. Furthermore, like other member countries of the Organisation for Economic Co-operation and Development (OECD) [21], the Netherlands is experimenting with payment reforms that have received considerable scholarly attention (e.g., [22,23]). This paper outlines the discourses encompassing integrated payment and its argumentative rationalities, thereby contributing by expanding our understanding of how integrated payment progresses, and why it is pursued or slowed down.

## 2. Research Materials and Methods

### 2.1. Materials

This discourse analysis focusses on integrated payment in the Netherlands. It is in communicating and discussing policy that the values and beliefs of stakeholders come to the fore [24]. The primary stakeholders in the policy debate on integrated payment are the political-administrative system, interest groups, providers, and insurers. The political-administrative system is defined as consisting of the Ministry of Health, Welfare and Sport (VWS), two regulatory bodies: the Dutch Healthcare Authority (NZa) and the National Health Care Institute (ZIN), and the Dutch House of Representatives. Interest groups consist of professional and advocacy associations.

To identify discourses on integrated payment by the various stakeholder groups, parliamentary databases were searched to identify relevant debates and letters from and to the Minister of Health, Welfare and Sport (henceforth: Minister). This search was supplemented with a scan of other policy reports, press releases, and letters on stakeholders’ websites and in newspaper and magazine articles retrieved from the Nexus Uni database. The search terms used were Dutch words or terms reflecting “integrated payment”. We analyzed documents covering the period from January 2009 to October 2021. The year 2009 was chosen as the start date because the NZa published a report on the feasibility of integrated payment for cardiovascular risk management (CVRM), diabetes, chronic obstructive pulmonary disease (COPD), and heart failure care in that year [25]. This report marked the beginning of the debate on integrated payment. Through this search process, a total of 89 documents were found (Table 1).

### 2.2. Methods

Discourses are defined as expressions, statements, and concepts used to frame how an object is understood and constructed [26,27]. Here, we apply a discourse analysis as it aims to understand how and what shared meanings—underlying values and beliefs—are conveyed through language by stakeholders [28,29]. The analysis provides politicians, policymakers, and practitioners with “frameworks for debating the value of one way of talking about reality over other ways” [26] (p. 5). Through illuminating distinct discourses over time, we can explore the “argumentative rationalities” [30] that stakeholders use to embrace or reject the implementation and use of an integrated payment policy, and hence its justification and viability going forward. To effectively illuminate the discourses, the associated materials are interrogated based on a heuristic consisting of four questions, loosely inspired by Bacchi [31]: For which problem is integrated payment the supposed solution? What are the underlying assumptions that justify or reject integrated payment as the solution? How is this solution advocated, questioned, or disputed by different stakeholders? And how do stakeholders’ argumentative rationalities change over time? These questions help to identify distinct discourses, and since a set of statements is rarely “watertight” [26], some overlap between discourses is acceptable.

### 2.3. Data Analysis

To analyze the data, the collected material was imported into ATLAS.ti. The analytical process consisted of two stages. First, documents were inductively coded [32]. During this phase, topics were manually linked to textual units such as phrases, sentences, or paragraphs. Codes were established that were as close as possible to the subject of the statement [33]. For example, the fragment “[X] is in favor of integral funding, but each healthcare provider now has a separate financial incentive that is not always in line with the interests of the pregnant woman” was coded as {incentives_X} and {collaboration_X}, where X is the relevant stakeholder. Codes were also labelled in terms of positivity or negativity towards integrated payment. Having created this set of codes, the remaining material was brought in to refine the codes. An extensive open coding process was followed by a thematic analysis in which the sets of codes and underlying data were assembled into groupings based on relationships between the codes to reveal wider patterns. In this phase, we were looking for codes that were related to each other. It was from these groupings of codes that the four discourses were constructed.

## 3. Results

The various stakeholders identified a range of problems in the current organization of and payment for healthcare for which they perceive integrated payment to be a solution. When announcing the new policy on integrated payment, the then Minister framed it as a paradigm shift:

*We are abandoning the idea on which our healthcare system was based for years: paying money to a hospital or a group of professionals. And we embrace the idea of where our healthcare system is heading in the coming years: paying money for a healthcare plan, for a complete treatment. So, we move from the who to the what. (emphasis added).*

[34]

The above quote illustrates that integrated payment is posited as a clear solution to an issue that is represented as being problematic and hence should be abandoned—integrated payment is necessary to move from fragmented to integrated care. This statement is also illustrative of the fact that the integrated payment policy was very much a top-down push, instigated by the Ministry and NZa and then widely discussed in Parliament and by other stakeholders. Identifying which discursive practices are elicited can reveal stakeholders’ argumentative rationalities. For instance, stakeholders might recognize the problem but criticize the solution for reasons of their own. These rationales are reflected in four distinct yet inter-related discourses: (1) Quality-of-Care, (2) Affordability, (3) Bureaucratization, and (4) Strategic.

### 3.1. Quality-of-Care Discourse

The discourse on quality of care has been multifaceted and is based primarily on the assumption that introducing integrated payment is a possible way to improve collaboration and quality of care. The Minister articulated this as follows:

*Health insurers can purchase care integrally and care providers are encouraged to collaborate in integrated care arrangements that are designed around the demand for care. By stimulating collaboration in healthcare, the quality of healthcare can be improved.*

[35]

However, not all stakeholders agreed with this reasoning, stressing not the potential for collaboration, but rather the necessity to improve care quality through patient-centeredness. The NZa emphasized that the existing payment system was overly provider-focused, implicitly assuming that integrated payment would shift the focus away from the provider and toward the care recipient. In doing so, they allude to possible quality improvements:

*Care should be organized around the patient, not the patient around the care. This applies in particular to the method of payment, which is currently focused too much on the provider and not on the care recipient. One possible way to achieve better quality and affordable primary care is the introduction of integrated payment.*

[25]

From the above quote, we can see that the NZa is alluding to “a possibility” to improve primary care (here in the context of COPD, CVRM, diabetes)—something that seems to contradict a later statement by the NZa concerning maternity care. That is, when the Royal Dutch Organization of Midwives (KNOV) emphasized that the assumption that integrated payment would lead to better quality had not been demonstrated, this position was endorsed by the NZa, as illustrated in the following quote:

*[Integrated payment] does not enforce better and shared care. The KNOV professional group has expressed this aptly in a response to the NZa: ‘It is an illusion to think that improvements in healthcare are achieved through a different payment method or by choosing to accommodate all chain partners in one organization’.*

[36]

Later (in 2015 and 2021), this sentiment was repeated by the KNOV with support from another maternity care advocacy group and the Dutch Patient Federation, continuing to underline that evidence for quality improvements was lacking. While the NZa seems to be the only party to have rapidly embraced the “integrated payment leads to improved care” axiom, over time a shift is visible. From 2012 onwards, a group of stakeholders—including the Ministry, several insurers, the Dutch Society for Obstetrics and Gynecology (NVOG), the trade association of Dutch healthcare organizations (ActiZ), and the NZa—began to espouse the “integrated payment leads to improved collaboration” axiom. On one occasion this reasoning was also embraced by the KNOV, with a policy advisor being quoted as saying that integrated payment helps “in further improving cooperation and mutual trust” [37]. Further, the committee responsible for the evaluation of integrated payment for diabetes, CVRM, and COPD observed that adequate integrated care could also be provided without integrated payment.

The necessity and sufficiency of integrated payment as a determinant of certain proximal (e.g., better collaboration or fewer financial incentives) or final (e.g., improved care, patient-centeredness) outcomes is contested by a broad range of stakeholders. The NZa appeared to concur that integrated payment could be a final element rather than a precondition:

*Providers and insurers have stated that integrated payment can be the final element of the substantive improvements that are now being implemented, but not the start. The NZa agrees with this view. Payment is generally the final element and not the engine of the reorganization of the collaboration.*

[36]

Besides this, a range of stakeholders have addressed the role of incentives that stems from the current, siloed reimbursement system [38,39,40,41]. Not all go so far as the Minister in asserting that paying individual providers incentivizes providers to focus on keeping a patient in their own domain or organization:

*I expect that the fully-fledged option of integrated payment will offer many opportunities for collaboration between gynecologists, midwives, and maternity nurses who voluntarily opt for this. Within the current system of separate payments, this is much more difficult to achieve because there is an incentive to continue to treat pregnant women within their ‘own line’ (emphasis added).*

[42]

Once more, it is the Ministry which, in another report, tries to succinctly explain how exactly a form of integrated payment will contribute to better care:

*For example, by funding related healthcare activities based on integrated rates, it no longer matters to individual healthcare providers how many treatments they can claim themselves. Instead, healthcare providers are incentivized to organize healthcare as well as possible in collaboration with other providers in their network (emphasis added).*

[43]

Other parties deliver descriptive rather than causative statements when problematizing the role of incentives. For example, one political party indicated that each provider has a distinct financial incentive that is not always in line with pregnant women’s interests [39] and a gynecologist commented that integrated payment removes undesirable financial incentives [40].

### 3.2. Affordability Discourse

This discourse focuses on the value added by integrated payment in economic terms: will integrated payment guarantee affordability? Ensuring the long-term affordability of healthcare for future generations was one of the reasons given by the Ministry for implementing an integrated payment scheme for chronic diseases (diabetes, CVRM and COPD) in 2009, claiming that the increased prevalence of chronic diseases was an important factor in rising healthcare costs:

*According to the Minister, seventy percent of the total health insurance costs go to twenty percent of the insured: the chronically ill. Integrated care is necessary to guarantee the affordability of care in the future.*

[34]

As such, the Minister is claiming that integrated care is the mediating instrument through which integrated payment leads to affordability. There are numerous other underlying assumptions on how integrated payment affects affordability. Two such assumptions are that it reduces costs associated with duplicate services [25,44] and enables the possibility of shifting care from secondary to primary providers and also within primary care options [25,45]. By coordinating care, there is less likelihood of duplicating activities and, hence generating duplicate costs. The presumption that substitution would lead to savings is linked to the removal of functional barriers between providers:

*This last aspect of [integrated payment] will entail major cost savings. According to the CPZ [College for Perinatal Care], an obvious saving of millions of euros. Certainly, if the substitution from secondary to primary care is taken as the point of departure for this tariff structure, it is inevitable that costs will be saved with the introduction of the [integrated payment]. After all, the more expensive secondary care is partly being replaced by primary care.*

[45]

However, over time, part of this rationale has been increasingly questioned, and the reality of substitution disputed by the KNOV. According to them, rather than substitution, it is medicalization that is taking place. Medicalization, a process through which primary care is shifted to secondary care, is contrary to substitution from secondary to primary care. If substitution is assumed to lead to savings, then medicalization would presumably lead to cost increases, and for that reason negatively impact affordability [46].

Furthermore, the Minister claimed that integrated payment would serve as an instrument to empower healthcare insurers to invest in prevention measures through which “sudden exacerbations and complications of conditions—and the associated healthcare costs—can be reduced” [47]. Although the affordability discourse has been largely dominated by the Ministry and NZa, throughout the debate several parties have questioned whether there is any supporting evidence that integrated payment leads to cost reductions [48,49].

### 3.3. Bureaucratization Discourse

The bureaucratization discourse addresses bureaucratic practices and structures, problematizing rules and regulations. The legal bedrock of the Dutch healthcare system is constituted in four healthcare acts, each with its own regulations and consequent budgetary frameworks and budget areas. It is argued that, from the patient’s perspective, these budgetary frameworks form artificial financial “barriers” [50]. The Dutch Patient Federation asserted that is seemed plausible that integrated care requires integrated payment “across all barriers” [51]. This aligns with the Minister’s view, supposing that there is a need to remove barriers between secondary and primary care, between professional cultures, and between financial flows [52]. One political party, referring to the financial barriers that derive from three healthcare acts, espoused this as follows:

*ZIN proposes looking at integrated payment for dementia care. That money now comes from the Zvw [Health Insurance Act], AWBZ [current Long-term Care Act] and Wmo [Social Support Act] pots, my [political] party wants to remove those financial barriers so that integrated care and the dementia care standard can really get off the ground.*

[53]

Hence, the implication is that removing financial barriers by integrating payment flows would propel integrated care. In the same spirit, the Minister argues that “separate funding can lead to treatment in the wrong place, by the wrong provider” [54]. Overall, there appears a broad consensus that separate funding and financial barriers are an obstacle to integrated care. As such, integrated payment is an instrument to transcend paywalls or merge cash flows in the “current, sector-based funding system” [42] and would ease navigating the rules of the, now fragmented, system.

Another consequence of rules and regulations is an administrative burden experienced by healthcare professionals. Integrated payment could potentially reduce the administrative burden, with stakeholders ascribing qualities such as clarity and uniformity to it [55,56]. A policy advisor working in the field of rehabilitation care (ActiZ) gave the following example:

*There is no funding within medical care for [some] consultations such as the multidisciplinary consultations for specific patient groups. If you work together as an interdisciplinary team, you need an integrated payment. From some form of money package, you should be able to see what you need for a specific patient without having to [go] through all sorts of detours and troubles to identify which [billing code] you can [apply].*

[56]

This policy advisor is emphasizing that integrated payment would remove the cumbersome efforts that the current system demands. However, this viewpoint, that an integrated payment is associated with less bureaucratic practices and administrative hassle is not embraced by all. In an opposing view, a group of stakeholders—consisting of an insurer, political parties, an interest organization, and an integrated maternity care organization (IMCO)—argued that integrated payment amplifies rather than reduces administrative complexity. Already by 2011, an insurer was quoted as saying that integrated payment for CVRM, COPD, and diabetes had led to additional bureaucracy that made the administrative complexity of the previous reimbursement system based on diagnosis-treatment combinations pale in comparison. The insurer continued as follows:

*It is not a reassuring thought that, in the future, all these integrated care arrangements will only be funded through an integrated payment. After all, it concerns ever-changing partnerships of care providers, some of which have already been corrected for overhead costs, while others have not. [Administrative] cleaning problems and duplication of healthcare costs will soon be the order of the day.*

[57]

The claimed increase in administrative complexity seems to be mainly because of the increasing number of agreements involving changing constellations of parties. Similarly, Bo Geboortezorg, the advocacy group for maternity nurses and care, argues that IMCOs will face such complexity:

*This current form of [integrated] payment, in which maternity care organizations in many different IMCOs have to deal with all kinds of different agreements, is unworkable. The obstacles, imperfections, and undesirable effects are so big that we no longer see any benefit in it.*

[58]

Another factor adding to the administrative complexity is the prospect of lingering duality. When integrated payment was introduced, the current payment models were retained. A politician raised the question of how these two modes can co-exist and what the bureaucratic implications would be:

*Two reimbursement systems, what will they yield for bureaucracy? Will there be multiple contracts within a region? What substantive requirements will the health insurer set for integrated payment? What if a pregnant woman wants to make other choices than those the birth center can offer her, for example a different midwife from a practice that is not affiliated, or another hospital? That’s not going to work […]. A pregnant woman has something else on her mind than those worries.*

[59]

It is important to emphasize that, according to this political party’s logic, not only would the provider and the insurer fall prey to increased bureaucracy, but also the new payment model could ultimately disadvantage the patient.

### 3.4. Strategic Discourse

The strategic discourse is dominated by those who argue that the power dynamics created by the integrated payment system are disadvantageous to the care process and its outcomes. Already in 2009, the Ministry was recognizing these dynamics, emphasizing that “working with integrated payment requires a certain development of the market relationships between main and subcontractors in the negotiation process and it entails new uncertainties for individual providers” [60].

The new dynamic between providers as main contractors and as subcontractors was viewed as undesirable by one insurer [61]. To them, the expansion of the integrated payment model was a system change that implied that care groups were given control over care at the expense of insurers. The insurer was worried about a loss of control over its purchasing activities, warning that the contracting between individual providers within care groups would become more important than the provider–insurer contracting [61]. This sentiment was echoed by a parliamentarian: “In reality, it is about who manages the payment and thus has power over the entire care process” [59]. Devolving the negotiation process from the insurer–provider dyad to the provider–provider dyad would furthermore distract from the care process and providing the appropriate care, instead encouraging discussions about who gets what. The statements below show that this latter point was raised by political parties in 2010 (concerning COPD, CVRM, and diabetes) as well as in 2021 (on maternity care).

*In practice, a general practitioner is now a contractor or subcontractor of a care group and must negotiate rates, whereby the price can be the main focus and not the quality. These members feel that this is at odds with establishing cooperation between care providers. Does this situation improve the quality of care?*

[48]

*Why are we so concerned with integrated payment? Who actually wants that? If you throw the [payment model] over the fence—because that’s what happens—then it is placed with the midwife and the [medical] specialist. They then have to negotiate about who gets which part of the financial pie. Surely that has nothing to do with good care, where everyone contributes what is needed from their own professionalism? […] Now, it is still the case that if one gets more, the other gets less.*

[62]

Besides the implications for what integrated payment would have for the negotiation process itself, stakeholders held assumptions about the consequences that would arise after the negotiation process. There was a belief that an integrated payment is an “instrument” that wields power to those who control it, as articulated by a political party thus:

*It is obvious that the current financing system has perverse incentives. That is also noted. The question is, however, what the outcome should be. Are we introducing a completely new system of integrated payment, in which one party, i.e., the hospital, the gynecologists, will probably be in the lead? That is the threatening reality. Or can we not take away those one or two perverse incentives and solve it in a different way?*

[59]

In the same spirit, another political party perceived a risk that community midwives would become subcontractors of the hospital if the insurer decided that the “pot of money” should be given to the hospital [59]. The concerns over the threat that integrated payment would supposedly pose to community midwifery were repeatedly voiced by various parties. Various political parties and the KNOV argued that with integrated payment, community midwives within IMCOs would be dominated by hospitals [47,62,63]. The Dutch Organization of Midwives and Pregnant Women (NOVEZ) believed that integrated payment would lead to the disappearance of community midwifery, “as a result of which hospitalization and medicalization, and with it the costs of care, will increase at a rapid pace” [64]. In line with this, political parties also signaled that integrated payment could harm the professional autonomy of community midwives and patients’ freedom of choice:

*The professional autonomy of the midwife and the continued existence of the independent practice—and thus the woman’s freedom of choice to give birth at home in a familiar and peaceful environment—are at stake due to the integrated payment policy rule.*

[65]

Another political party considered it important to come to a form of payment that did justice to the interests of all the parties involved, and primarily those of pregnant women [59]. The main argumentation in this discourse was focused on highlighting that integrated payment would reshuffle the positions of parties in the negotiation process, the belief that it would have negative effects on the professional autonomy of the midwife and the freedom of choice of the patient, and that it would lead to increased medicalization. These rationalities were countered in several ways by the Ministry. First, it was argued that medicalization decreased in IMCOs that used integrated payment [59]. Second, it was asserted that IMCOs would presumably have an incentive to organize the care further upstream:

*For each pregnant woman, the integrated tariff will be paid to the maternity care organization, so the organization will also have an incentive to organize care ‘as low as possible’. In my view, integrated funding offers opportunities for midwives to strengthen their position in maternity care.*

[42]

That is, contrary to what had previously been argued by others, this development would reinforce the position of community midwifery because care would be rearranged within secondary care, or shifted from secondary to primary care. Further, the underlying assumption that primary care is more economic than secondary care would increase the likelihood of savings at the behest of IMCOs. Third, concerning pregnant women’s freedom of choice, the Minister assured doubters that a pregnant woman would retain the freedom to choose caregivers from other IMCOs: “switching to another network is possible” [42], although this might complicate the payment modality as we saw in the previous discourse.

## 4. Discussion

This discourse analysis set out to gain insight into the justification and viability of continuing the implementation of integrated payment in the future by determining which discourses predominate, how they have changed over time, and how they differ among key stakeholders. Of the four discourses identified, the discourses on Quality-of-Care and Affordability were present from the outset, reflecting the justification for introducing integrated payment: that it will improve the quality and affordability of care. As time has moved on, Strategic and Bureaucratization discourses have come to the fore because the implementation process has exposed the consequences of integrated payment in terms of power, interests, and administrative burden. Furthermore, we have shown that key stakeholders hold different positions within various discourses: whereas policymakers and regulatory bodies tend to take a positive stance toward integrated payment, those involved in carrying out care, such as providers, their advocacy organizations, and healthcare insurers, tend to be more skeptical of the payment reforms.

In the transition from traditional to integrated payment models, the notion was put forward that “the old is dying but the new cannot be born” [66] (p. 276). This was because this phase was accompanied not only by resistance from stakeholders but also with “symptoms” such as increased bureaucracy and an overall lack of clarity as to where the system was heading. At the same time, fragmented ways of paying, such as fee-for-service and diagnosis-treatment combinations, remain necessary for two reasons: not all care is amenable to integration (one-off care), and integration leads to new fragmentation [67] prompting an integration–fragmentation tradeoff. Furthermore, traditional models should continue to function as a necessary, fundamental backbone until integrated payment models have proved able to achieve their objectives.

As such, solving the integrated payment puzzle can be seen as a “wicked problem”: actions oriented toward solving it typically have unintended consequences elsewhere in the system [68]. Our analysis indeed shows that aiming to solve issues pertaining to quality and affordability through proposing and implementing an integrated payment scheme has repercussions elsewhere. It has brought to the fore concerns about conflicting interests, the allocation of resources, and differences in power, status, and autonomy which, subsequently, if deemed desirable, will have to be smoothed through a variety of “reconciling mechanisms” making integrated payment even more diverse and complex [69]. Furthermore, these tensions will be amplified when integrated payment initiatives expand beyond their current scope and extend to the interface between health and social services [11]. As a consequence of this, even more parties with diverse backgrounds have to strategically interact and other traditional payment models will also have to be transformed and fused into integrated payment or financing arrangements.

In the strategic discourse, professional autonomy has proven to be one of the key concerns. Theoretically, an integrated payment scheme is credited with providing integrated delivery systems with flexible use of resources (i.e., money) [70] and also with expanding professional autonomy, both clinically and economically [71]. While some forms of integrated payment might indeed increase autonomy on the service delivery network level, the results of this study suggest that the professional autonomy of one provider vis-à-vis another is put under pressure. With the introduction of integrated payment, these networks are transformed into micropolitical economies in which individual actors seek to acquire the scarce resources necessary to sustain their activities [72]. Powerful actors can control the flow of these resources, thereby failing to utilize the potential benefit of deploying resources flexibly in order to optimize care. Another consequence is that less powerful actors struggle to maintain a claim on their professional activities [72], resulting in diminished professional autonomy. As such, policymakers and managers should be aware of the implications that integrated payment has on professional autonomy. Here, Ten Have [73] argues that a “scarcity of resources requires the development and implementation of strategies for the just distribution of resources”, concluding that “it is an institutional duty to develop fair mechanisms of allocation and selection” (p. 504), thereby emphasizing the moral-political aspect of the question “who is getting paid, how much, for doing what?” [74] (p. 7).

It is important to consider the role of research evidence in discourses. As experiments progress and the payment landscape changes, a growing body of evidence (e.g., [75,76,77,78]) finds its way into the policy debate. The opportunity to use research evidence to back partisan assumptions, interests, or beliefs increases as the evidence base continues to grow. However, more evidence does not necessarily lead to an evidence-based discourse [79]. While this discourse analysis has highlighted where, in some instances, stakeholders do point to evidence, or a lack thereof, to support their statements, it was not possible to conclude whether stakeholders willfully refute or disregard evidence that is not congenial to their interests.

Comparable developments in the field of payment policy can be observed in other OECD countries [21,80], and it is relevant to consider the contextual differences between countries including in who pays for care. The Netherlands has a multipayer system, in which comprehensive healthcare coverage is mandated by the government and subsequently offered by a number of competing, nationwide, insurers—similar to the systems applied in Austria, Belgium, Germany, Israel, and Switzerland [81]. Within regulatory boundaries, insurers are free to pursue their own purchasing strategies, which may include integrated payment. However, multi-payer systems are characterized by a lack of monopsony power, limiting the ability or desire of payers to push for novel payment policies [82]. The dynamics in competitive or other market forces [83] might affect the discursive mechanisms in multi-payer systems differently than in single-payer systems. Furthermore, these mechanisms might be affected by the differences in the laws and regulations present in other systems. We would therefore encourage investigation of the discourses on integrated payment schemes in other healthcare systems or regions.

Finally, our analysis revealed that the main actors in the discourses on integrated payment are the Ministry of Health, the healthcare authority NZa, political parties, insurers, care providers, and professional associations. Notably, patient advocacy organizations (PAOs) are absent from the discursive material. Although the role of PAOs in policymaking is widely recognized [84,85], the involvement of PAOs in payment reform initiatives and policy has not been acknowledged. Further research should therefore address whether, and if not, why not, PAOs are involved in payment reform because patients should be the ultimate beneficiary of any payment reform.

## 5. Conclusions

This analysis has identified four discourses on the values and beliefs surrounding integrated payment schemes. The future viability of integrated payment models will depend on how these models address issues concerning Bureaucratization and those coming to the fore in the Strategic discourse: issues of power, status, autonomy, and diverging interests. When addressing these issues, the tensions between the Strategic and Bureaucratization discourses on the one hand and the Quality-of-Care and Affordability discourses on the other will need to be carefully considered by policymakers, providers, and purchasers. The quality of care and its affordability are both important public interests in the Dutch healthcare system, and these should not be overlooked at the expense of bureaucratization and strategic issues.

It is reasonable to assume that the complexity surrounding the implementation of integrated payment systems will intensify due to an ever-increasing number of organizations becoming involved in further integrated payment initiatives, especially since this approach is expected to extend beyond the health domain to include the interface between health and other social services. Government has a stewardship role [86] and should nurture preconditions for pioneers to experiment with integrated payment. Accordingly, healthcare insurers—in their role of purchasers of care—should prepare and align their internal organization for future integrated payment initiatives, and providers should ensure a fair and just allocation of funds within the group, so that every practitioner sees the benefits of integrated payment.

## Figures and Tables

**Table 1:** Overview of documents used in the analysis.

Output Type	Number of Documents
Parliamentary papers	57
Reports	13
Professional magazine articles	8
Press releases and letters	8
Local/regional newspaper articles	3

## Data Availability

No new data were created or analyzed in this study. Data sharing is not applicable to this article.
